# Flow Diverter Stents for the Endovascular Treatment of Ruptured Intracranial Aneurysms: An Analysis of a Case Series

**DOI:** 10.7759/cureus.99412

**Published:** 2025-12-16

**Authors:** Jorge A Santos-Franco, Erika E Gonzalez-Sansores, Ricardo E Jiménez-Gatica, Alan R Campos-Cruz, Juan P Alonso del Toro, Miriam Lara-Muñoz

**Affiliations:** 1 Department of Neurosurgery and Neurological Endovascular Therapy, Specialty Hospital, La Raza National Medical Center, Mexican Social Security Institute, Mexico City, MEX

**Keywords:** endovascular treatment, flow diverter, rebleeding, ruptured intracranial aneurysm, subarachnoid hemorrhage

## Abstract

Objective

This study aimed to describe the clinical outcomes, complications, and aneurysm occlusion of patients with aneurysmal subarachnoid hemorrhage treated with a flow diverter in our institute.

Methods

This is a retrospective study of the clinical and imaging characteristics in patients with aneurysmal subarachnoid hemorrhage treated by endovascular approach using a flow diverter device from January 2019 to May 2024 at the Specialty Hospital, La Raza National Medical Center, Mexico. The information was obtained through the analysis of clinical records and imaging.

Imaging controls were obtained through digital subtraction angiography (DSA) and/or CT angiography (CTA) performed during the 12 months post-treatment. For statistical analysis, IBM SPSS Statistics for Windows, Version 25.0 (IBM Corp., Armonk, New York, United States), was used, with normality assessed using the Shapiro-Wilk test. Data were expressed as absolute frequencies, percentages, mean, and standard deviation. Additionally, the results are presented in descriptive tables for each patient.

Results

Fifteen patients were analyzed, of whom 73.3% were women and 26.7% were men. No patient experienced rebleeding or died during the 12-month follow-up period. On the 12-month follow-up imaging studies, 14 patients (93.3%) showed complete aneurysm occlusion. Three patients (20%) presented parent vessel occlusion associated with the flow diverter. Thirteen patients (86.7%) had a favorable functional outcome (modified Rankin scale (mRS) 0-2).

Conclusions

The use of flow diverters in patients with ruptured intracranial aneurysms can be considered an effective and viable alternative in carefully selected cases. Prospective studies with larger sample sizes are needed to evaluate their efficacy and safety.

## Introduction

Subarachnoid hemorrhage (SAH) is a medical emergency that requires immediate attention. Globally, non-traumatic SAH is caused by the rupture of an intracranial aneurysm in 85% of cases, accounting for 2-5% of all strokes, with an estimated global incidence of nine per 100,000 people per year [1] and a global prevalence of 8.09 million cases [2]. In Mexico, according to the RENAMEVASC study, it accounts for 12% of all patients presenting with stroke [3]. Of all patients presenting with SAH, approximately 43% experience significant morbidity, even though the various treatment modalities for the causative aneurysm carry a risk of about 6% of disability and death [1].

Among the early complications associated with SAH is rebleeding, with an incidence of 8-23% within the first 72 hours and up to 50% at six months [1], which is why the American Heart Association/American Stroke Association (AHA/ASA) 2023 guidelines recommend performing treatment as soon as possible, ideally within the first 24 hours [2]. Hydrocephalus is a complication that occurs in 15-87% of patients in the acute stage, of whom 8.9-48% will develop chronic hydrocephalus dependent on ventricular shunting. Delayed ischemic deficit caused by vasospasm is a complication that occurs in approximately 30% of patients between four and 14 days after SAH [2,3]. 

Regarding endovascular treatment, there are several options which can be grouped into (a) intrasaccular treatments, (b) intraluminal treatments (exclusively in the parent vessel), and (c) a combination of both [4,5]. The choice of endovascular treatment depends on the aneurysm's location and morphological features, such as fusiform or dissecting configuration, wide neck (>4 mm), and size (diameter ≥12 mm), as well as patient-specific factors. These include recent use of antiplatelet or anticoagulant agents, or the presence of a coagulation disorder, particularly in settings where the clinician does not have full access to reversal agents [4-6].

Flow diverters have become a powerful tool in the treatment of cerebral aneurysms. Traditionally, treatment with flow diverters in SAH is preferred to be avoided for various reasons: First, the need to initiate antiplatelet therapy increases the risk of rebleeding, and it also poses a limitation by raising the risk of bleeding in patients who may require subsequent surgical treatment, such as ventricular shunting, decompressive craniotomy, tracheostomy, etc. [7,8]. Second is the risk of rebleeding despite treatment, as aneurysm thrombosis usually does not occur immediately. Lastly, the prothrombotic phase in SAH can lead to a high rate of thromboembolic complications with the use of intraluminal devices [7,8].

However, there are cases in which, given the morphological characteristics of the aneurysm or patient conditions, treatment with flow diverters is necessary in aneurysmal SAH. For these reasons, various studies have been conducted, whose results suggest that the use of flow diverters in patients with ruptured aneurysms is feasible and safe [1,9]. 

Flow diverter device

The term diverter was first used in 2004 at the University of Miami during the study of low-porosity stents as a treatment for intracranial aneurysms [10,11]. The term "flow diversion" refers to an endoluminal remodeling of the vessel to divert intra-aneurysmal flow to promote (a) blood stasis within the aneurysm, (b) reduction of flow velocities, and (c) reduction of shear forces, all of which lead to eventual aneurysm thrombosis [12].

Therefore, the mechanism of the flow diverter in the treatment of the intracranial aneurysms involves three phases: (1) The hemodynamic phase occurs immediately after the procedure, due to the layers of the flow diverter component that lead to the obstruction of blood flow into the aneurysm. (2) The thrombosis phase is a consequence of the reduction of intra-aneurysmatic arterial flow, which in turn leads to a decrease in flow velocity within the aneurysm, followed by platelet activation and progressive thrombus formation over days to weeks. (3) The endothelialization phase involves the transformation of the thrombus into collagen. This stage can last months to years and leads to a reduction in the aneurysm volume [11,13,14]. 

Notable clinical trials evaluate the utility and risks of flow diverters in the treatment of ruptured cerebral aneurysms. Natarajan et al. published in 2017 the results of 11 patients with SAH treated with a flow diverter, showing a good functional recovery (modified Rankin scale (mRS) 0-2) in 81.8% patients at 30 days and a mortality rate of 18.8% at 30 days. In the same series, 100% of the aneurysms were obliterated at approximately 18 months of follow-up [8]. Cohen et al. published in 2021 the results of 76 patients, reporting a 7.9% rate of procedure-related complications, of which 2% had permanent neurological damage. None of the patients experienced aneurysm rebleeding. The occlusion rate was 95.5% in the 12-month follow-up, with 6.6% dying during follow-up due to causes unrelated to the procedure [10]. Likewise, Gopinathan et al. in 2021 published a series of 22 patients with a follow-up of 8.5 months, reporting three patients with procedure-related complications; no rebleeding was reported during follow-up [1]. The clinical outcome was good (mRS 0-2) in 86.3% of patients; three patients died due to SAH complications unrelated to the procedure. As for Rantamo et al., in 2024, they published their findings on the treatment of 39 patients, reporting 37-42% of ischemic complications, a 30-33% incidence of hemorrhagic complications, and an 11% rate of rebleeding [9]. Ten Brinck et al. conducted a systematic review between the years 2010 and 2021 involving 26 patients with SAH treated within the first 15 days of clinical presentation. This publication included 26 retrospective studies with a total of 367 patients. The analysis revealed that 85.6% of the cases demonstrated complete aneurysm occlusion and 73.7% had a favorable neurological outcome (mRS 0-2), while the mortality rate was 17.1%. Only 3% registered rebleeding. The authors highlighted that patients with a poor clinical presentation (Hunt and Hess grade 4) and large or giant aneurysms were associated with a worse prognosis [7].

## Materials and methods

This is a case series including all patients with aneurysmal SAH treated with a flow diverter between January 2019 and May 2024 at the Specialty Hospital, La Raza National Medical Center, Mexico. The study was approved by the Institutional Ethics Committee of the Mexican Social Security Institute (approval number: R-2025-3501-126).** **

The primary objective was to describe the degree of aneurysm occlusion, the frequency of rebleeding, and the functional outcomes of patients with ruptured intracranial aneurysms treated with a flow diverter at our institute. The secondary objectives were as follows: to describe the degree of occlusion of ruptured intracranial aneurysms treated with a flow diverter, to determine the frequency of complications related to flow diverter implantation, and to evaluate the functional clinical evolution of patients at the 12-month follow-up. Clinical and imaging records of patients with aneurysmal SAH treated with a flow diverter between January 2019 and May 2024 were reviewed.

Selection criteria

The inclusion criteria were as follows: (a) diagnosis of aneurysmal SAH, (b) age >18 years, (c) treatment with a flow diverter between January 2019 and December 2024, and (d) availability of CT angiography (CTA) and 12-month follow-up digital subtraction angiography (DSA). In contrast, the exclusion criteria were the following: (a) upper gastrointestinal bleeding, (b) contraindication to oral antiplatelet therapy, (c) treatment with surgical clipping, and (d) treatment with simple coiling or stent-assisted coiling.

**Figure 1:**
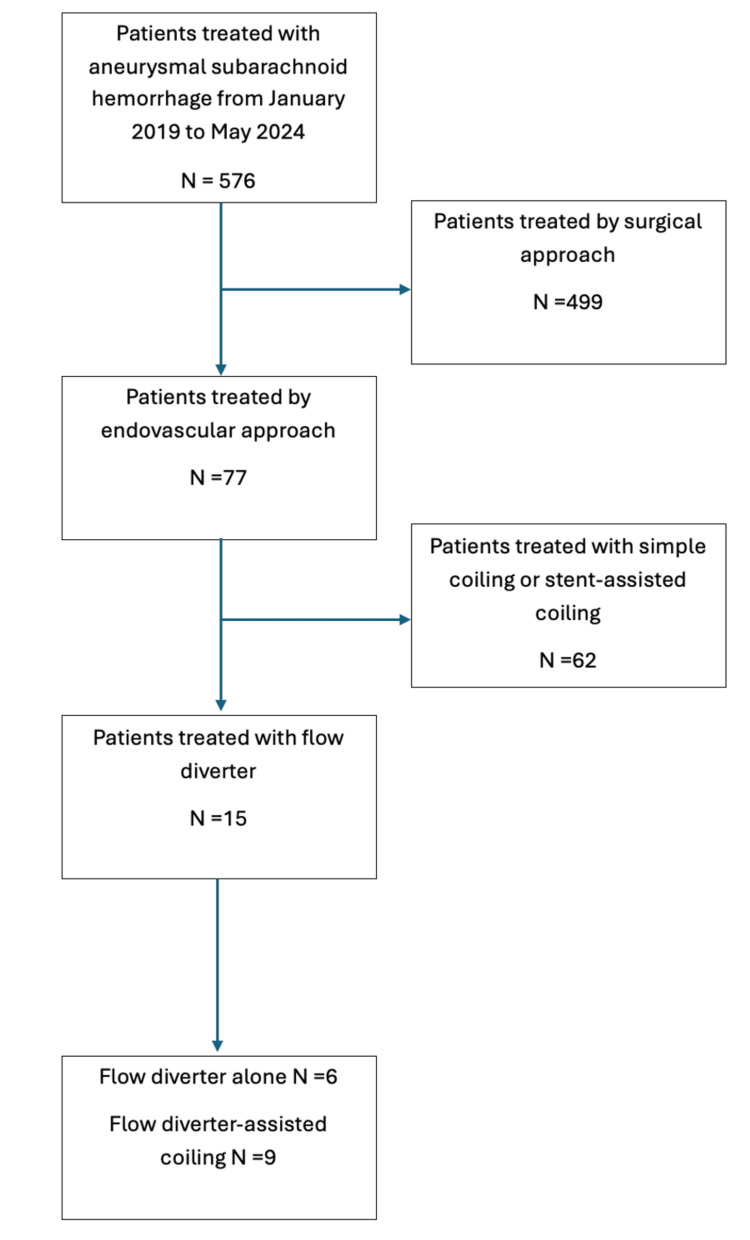
Study profile

A total of 576 patients with aneurysmal SAH were hospitalized; among them, 499 underwent surgical clipping, and 77 received endovascular treatment. Of these, 62 patients treated with simple coiling or stent-assisted coiling were excluded. Fifteen patients with aneurysmal SAH treated with flow diverters were included (Figure 1).

Clinical information was obtained from medical records. Clinical severity at admission was assessed using the Hunt and Hess scale [15], and radiological severity was evaluated using the Fisher scale [16]. Patients presenting with hydrocephalus at admission were treated with ventricular shunting prior to the endovascular procedure.

Antiplatelet regimen

During the procedure, patients received an intravenous loading dose of tirofiban at 0.4 mcg/kg/min for 30 minutes, followed by a maintenance dose of 0.1 mcg/kg/min. At the end of the procedure, an oral loading dose of 300 mg acetylsalicylic acid and 300 mg clopidogrel was administered. Four hours after the oral loading dose, intravenous tirofiban was discontinued. Oral antiplatelet therapy was continued with acetylsalicylic acid 150 mg every 24 hours for 12 months and clopidogrel 75 mg every 24 hours for six months.

Endovascular techniques

During the initial diagnostic cerebral angiography, aneurysm morphology, location, size, and neck characteristics were assessed. Saccular aneurysms were defined as focal dilations or outpouchings that did not involve the entire circumference of the vessel. Fusiform aneurysms were defined as those involving the full circumferential diameter of the parent vessel. Dissecting aneurysms were identified by the presence of an intimal flap, double lumen, focal enlargement, irregular mural dilations, or segmental/irregular stenosis.

Aneurysms were classified as wide-necked if the neck measured ≥4 mm or the dome/neck ratio was <1.5. All aneurysm dimensions were recorded in millimeters, and aneurysm location was documented. Two endovascular treatment strategies were used: flow diverter alone and flow diverter-assisted coiling. Flow diverter-assisted coiling was performed for aneurysms ≥12 mm or those presenting with a saccular morphology. The choice of flow diverter device was left to the discretion of the treating physician.

Follow-up

Clinical evaluations were performed at three, six, and 12 months. Imaging follow-up consisted of CT angiography at six months and DSA or CT angiography at 12 months. A favorable clinical outcome was defined as an mRS score of 0-2 [17]. Complications were assessed during follow-up and classified as related or unrelated to flow diverter implantation or endovascular technique. The degree of aneurysm occlusion was evaluated using the O'Kelly-Marotta (OKM) scale [18]. Patency of the parent vessel was also assessed.

Statistical analysis

Statistical analysis was performed using IBM SPSS Statistics for Windows, Version 25.0 (IBM Corp., Armonk, New York, United States). The Shapiro-Wilk test demonstrated normal data distribution. Nominal qualitative variables were expressed as absolute frequencies and percentages. Continuous quantitative variables were reported as mean±standard deviation. Results are presented in descriptive tables for each patient.

## Results

In our study, females represented 73.3% of the cohort, while males accounted for 26.7%. The age of the patients ranged from 22 to 81 years, with a mean age of 55.8 years (SD 16.64).

Clinical severity at admission, according to the Hunt and Hess scale [15], was grade 2 in 53.3%, grade 1 in 6.7%, and grade 3 in 40% of patients. Hemorrhage severity based on the Fisher scale [16] was grade 4 in 73.3% and grade 3 in 26.7% of cases. Hydrocephalus was present at admission in 26.7% of patients; these individuals underwent ventricular shunting prior to the endovascular procedure.

Aneurysm morphology was classified as follows: saccular in 73.3%, fusiform in 13.3%, and dissecting in 13.3% of patients. The diagnosis of dissecting aneurysms was angiographically confirmed by the presence of an intimal flap, double lumen, focal enlargement, irregular mural dilations, or segmental/irregular stenosis. All patients with dissecting aneurysms achieved complete occlusion without neurological sequelae.

The time from symptom onset to endovascular treatment had a mean of 14.13 days (SD 7.15; range 5-28 days).

**Figure 2:**
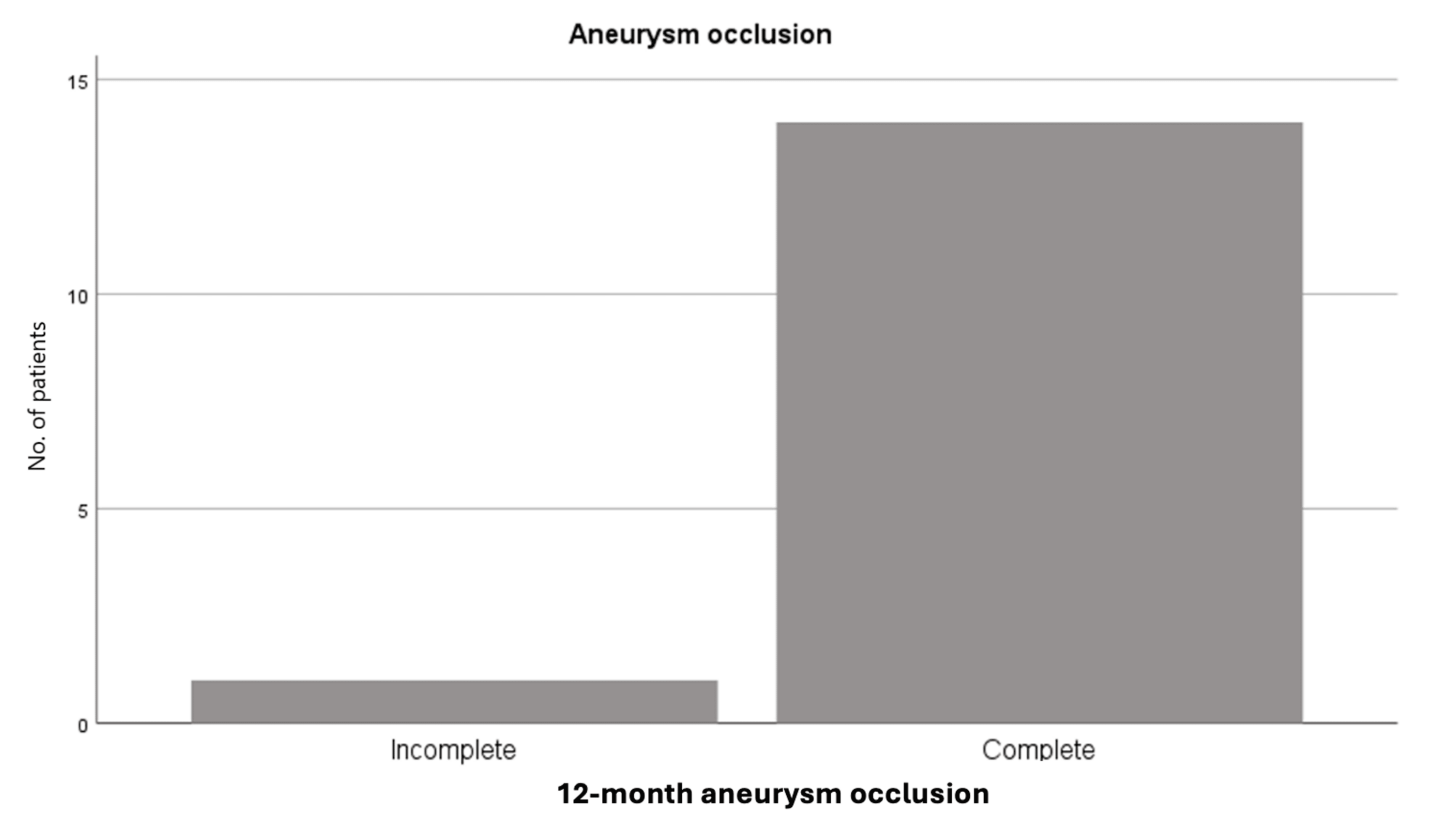
Frequency of aneurysm occlusion at the 12-month follow-up

Two endovascular treatment strategies were used: (1) flow diverter deployment alone, performed in six patients (40%), and (2) flow diverter-assisted coiling in nine patients (60%). Regarding aneurysm occlusion, according to the OKM scale [18], 14 patients (93.3%) demonstrated grade D occlusion at both six- and 12-month follow-up. One patient remained at grade A and subsequently underwent successful surgical clipping (Figure 2).

**Figure 3:**
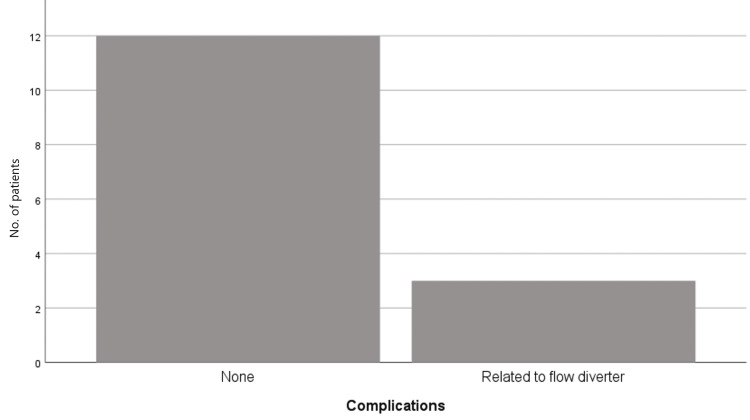
Frequency of complications related to the treatment with a flow diverter in our center

During the follow-up period, three patients (20%) experienced parent artery occlusion as a complication associated with flow diverter use. This occurred due to non-adherence to the prescribed antiplatelet regimen after hospital discharge. Among these patients, Patient 11 developed aphasia and hemiparesis, resulting in an mRS score of 4. Patients 4 and 8 had sufficient collateral circulation and did not exhibit new neurological deficits beyond those present at admission (Figure 3).

Complications not related to flow diverter implantation included hydrocephalus, vasospasm, and delayed cerebral ischemia. Patient 3 had an mRS score of 3 due to dense hemiparesis secondary to delayed cerebral ischemia. Patient 10 experienced hydrocephalus and vasospasm as complications unrelated to flow diverter use, with an mRS score of 2 due to mild hemiparesis resulting from cerebral vasospasm.

**Figure 4:**
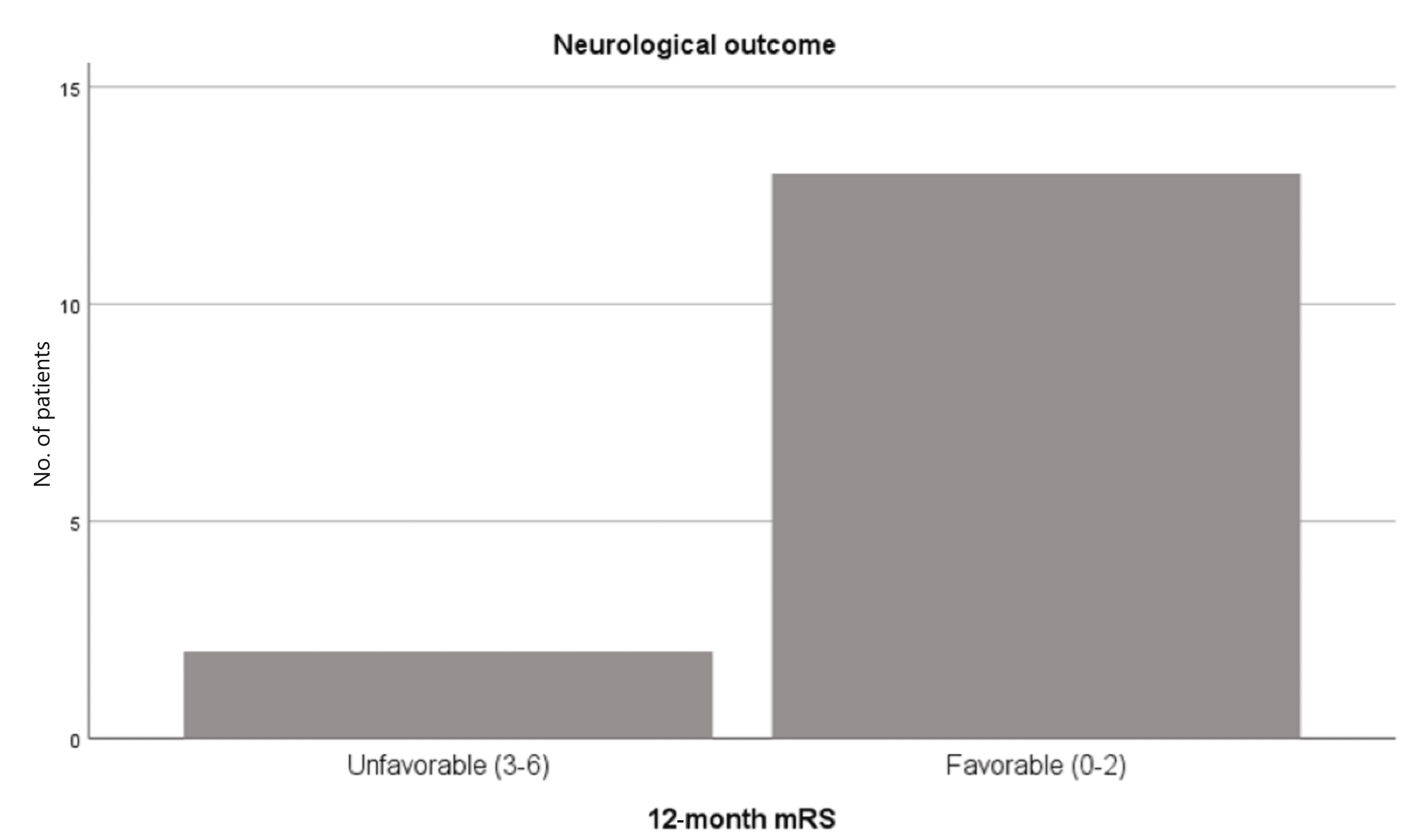
Neurological outcomes according to the mRS in aneurysmal subarachnoid hemorrhage patients treated with flow diversion mRS: modified Rankin scale

No rebleeding occurred during follow-up. Regarding functional outcome, it was favorable (mRS 0-2) in 13 patients (86.7%) and unfavorable (mRS 3-6) in two patients (13.3%) [17]. None of the patients died (Figure 4).

The results are described in Table 1.

**Table 1:** Clinical and treatment characteristics of all patients included in this study NA: not applicable; ICA: internal carotid artery; R: right; L: left; F: female; M: male; FD: flow diverter; C: coiling; FD+C: flow diverter-assisted coiling; OKM: O'Kelly-Marotta; mRS: modified Rankin scale; FRED: Flow-Redirection Endoluminal Device

Patient	Sex	Age (y)	Aneurysm location	Side	Aneurysm type	Maximum diameter of aneurysm (mm)	Fisher grade [16]	Hunt and Hess grade [15]	Days from aneurysm rupture to treatment	FD type	Technique	Aneurysm rebleeding	Complications not associated with FD use	Complications associated with FD use	mRS score on the last follow-up [17]	OKM scale [18]		Aneurysm obliteration at the 12-month follow-up
																Day 0	12-month follow-up	
1	F	49	ICA: paraclinoid	L	Saccular	17	4	1	16	FRED	FD+C	No	None	None	0	A2	D	Complete
2	F	22	ICA: communicating	L	Saccular	10	4	2	12	FRED	FD+C	No	Hydrocephalus	None	0	C2	D	Complete
3	M	38	Middle cerebral artery: M1	R	Saccular	18	3	3	23	Silk	FD+C	No	Vasospasm, delayed cerebral ischemia	None	3	A1	A1	Incomplete
4	F	60	ICA: choroidal	L	Saccular	16	4	2	8	FRED	FD+C	No	None	Embolic occlusion of the parental vessel	1	C2	D	Complete
5	F	62	ICA: paraophthalmic	R	Fusiform	10	3	2	7	FRED	FD+C	No	Vasospasm	None	1	B2	D	Complete
6	M	37	ICA: ophthalmic	R	Saccular	14	4	3	11	FRED	FD+C	No	Vasospasm	None	1	C3	D	Complete
7	F	72	ICA: ophthalmic	R	Saccular	6	3	2	9	FRED	FD	No	None	None	0	A3	D	Complete
8	F	62	Middle cerebral artery: M1	L	Fusiform	12	4	3	28	FRED	FD+C	No	Vasospasm	Embolic occlusion of the parental vessel	1	A1	D	Complete
9	F	81	Anterior communicating artery	NA	Saccular	4.7	4	3	11	FRED	FD	No	Hydrocephalus	None	1	A1	D	Complete
10	M	64	ICA: ophthalmic	R	Saccular	18	4	3	12	p64	FD+C	No	Hydrocephalus, vasospasm	None	2	C3	D	Complete
11	F	75	ICA: ophthalmic; ICA: clinoid	L	Saccular	12	4	2	14	FRED	FD+C	No	None	Embolic occlusion of the parental vessel	4	C3	D	Complete
12	M	42	Anterior communicating artery	NA	Saccular	4	4	3	26	p64	FD	No	Hydrocephalus	None	1	A2	D	Complete
13	F	49	Vertebral artery: V4	L	Dissecting	6	4	2	5	Silk	FD	No	None	None	0	A2	D	Complete
14	F	74	ICA: communicating	L	Saccular	4	4	2	21	Pipeline	FD	No	None	None	0	C3	D	Complete
15	F	51	Vertebral artery: V4	L	Dissecting	6	3	2	9	Pipeline	FD	No	None	None	0	C3	D	Complete

## Discussion

SAH due to intracranial aneurysm rupture is a medical emergency; therefore, early treatment is essential. Evidence shows that in selected patients with favorable aneurysm morphology, an endovascular approach is the preferred treatment, often requiring intraluminal devices such as flow diverters. However, their use invariably necessitates antiplatelet therapy, which, in the context of rupture, represents a significant challenge or limitation [1].

In this study, most aneurysms were saccular in morphology. Thirteen of the 15 patients included had ruptured aneurysms of the anterior circulation, whereas two patients had posterior circulation aneurysms. Both posterior circulation aneurysms were dissecting, confirmed angiographically by the presence of an intimal flap, double lumen, focal enlargement, irregular mural dilations, or segmental/irregular stenosis. These patients were treated with flow diverter placement alone and achieved complete occlusion with favorable functional outcomes (mRS 0), without complications. It is worth noting that these aneurysms measured approximately 6 mm in maximum diameter, which may have contributed to the absence of complications. Additionally, their treatment occurred at five and nine days after hemorrhage, earlier than the mean treatment time for the rest of the cohort.

The time from symptom onset to endovascular treatment had a mean of 14.13 days (SD 7.15; range 5-28 days). This delay in definitive treatment following hemorrhage is attributable to the fact that the study was conducted in a tertiary referral center, where patients are typically transferred from other hospitals approximately 5-15 days after the initial bleeding event.

**Figure 5:**
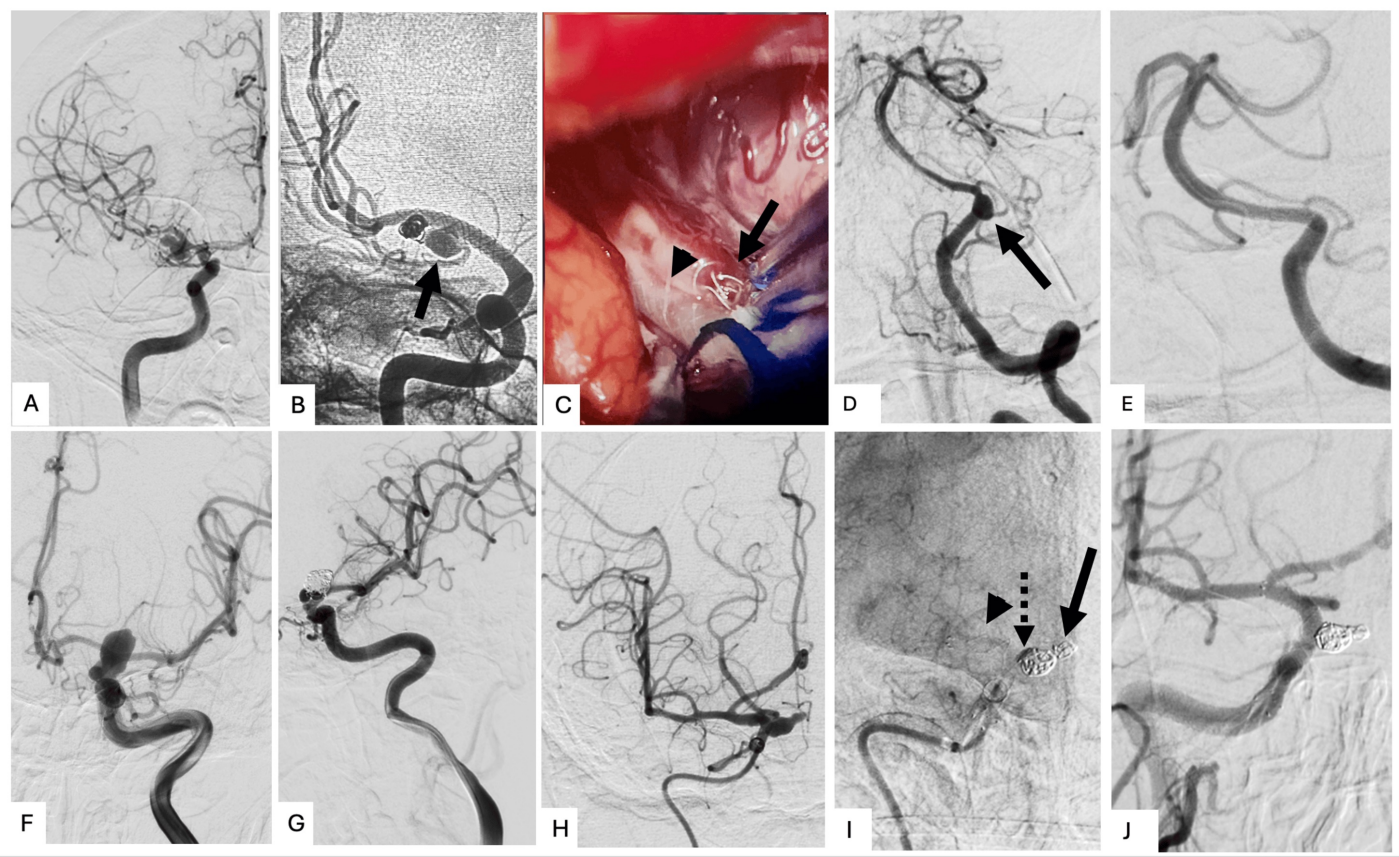
Representative cases Case no. 3: patient with aneurysmal subarachnoid hemorrhage treated with flow diverter-assisted coiling. (A) Angiography pretreatment. (B) Angiography at the 12-month follow-up, with partial aneurysm filling (arrow). (C) Intraoperative image of the aneurysm clipping that shows coils inside the aneurysmal sac (arrow), along with a flow diverter in the parent vessel (arrowhead). Case no. 13: patient with aneurysmal subarachnoid hemorrhage treated with a flow diverter alone. (D) Pretreatment angiography with a dissecting aneurysm (arrow). (E) 12-month follow-up angiography with aneurysm obliteration. Case no. 6: patient with aneurysmal subarachnoid hemorrhage treated with flow diverter-assisted coiling: (F) Pretreatment angiography. (G) 12-month follow-up angiography with aneurysm occlusion. Case no. 5: patient with aneurysmal subarachnoid hemorrhage treated with flow diverter-assisted coiling. (H) Pretreatment angiography. (I) Immediate post-treatment angiography without digital subtraction demonstrating coils (arrow) and flow diverter (arrowhead), with stagnation of blood inside the aneurysm (dotted arrow). (J) 12-month follow-up angiography with complete aneurysm occlusion.

Regarding treatment efficacy at 12 months, 14 patients (93.3%) achieved a favorable occlusion grade, with only one patient showing a residual aneurysm that subsequently required microsurgical treatment (Figure 5).

These results are consistent with those reported by Cohen et al. in 2021, who observed an occlusion rate of 95.5%, and with the findings of Ten Brinck et al. in their systematic review, which reported complete occlusion in 85.6% of patients [10].

In this study, hydrocephalus, vasospasm, and delayed cerebral ischemia were identified as complications not associated with flow diverter use. These events contributed to the functional outcomes of mRS 3 and 2 in Patients 3 and 10, respectively. Both patients presented Fisher grades 3 and 4 and Hunt and Hess grade 3 at admission, which is consistent with the expected clinical course of severe SAH. Given the small sample size, the proportion of patients with unfavorable outcomes may appear disproportionately high, although these events were unrelated to the flow diverter.

Hydrocephalus was observed in 27% of cases, comparable to the approximately 30% reported in the literature. Only patients who presented with imaging evidence of hydrocephalus at admission underwent ventricular shunting prior to endovascular treatment [2].

Regarding rebleeding, heterogeneous results have been reported. Gopinathan et al. reported no cases at 8.5 months of follow-up. Rantamo et al. documented a rebleeding frequency of 11%, while Ten Brinck et al.'s systematic review reported a frequency of 3%. In our study, no cases of rebleeding were observed. This finding may reflect a selection bias, as rebleeding typically occurs within the first 72 hours after rupture, and the patients in our series were treated after this high-risk period due to delays in referral to our center. Another factor to consider is aneurysm size: the aneurysms in our cohort had a mean diameter of 10.5 mm (range 4-18 mm). Aneurysms larger than 7 mm carry an increased risk of rupture; however, our results may also be influenced by the treatment modality used [7,10]. In the systematic review, the most common treatment strategy was flow diverter-assisted coiling, likely because saccular morphology predominated and coil placement provided dome protection while allowing the flow diverter to exert its reconstructive effect.

Regarding complications associated with flow diverters, parent vessel occlusion occurred in three patients in our series. Importantly, all cases were attributed to non-adherence to antiplatelet therapy during the first month after treatment. This rate is lower than that reported by Rantamo et al. in 2024, who described ischemic complications in 37-42% of cases [7].

Concerning functional outcomes, we observed a favorable mRS score (0-2) in 86.7% of patients, which is consistent with the findings of Natarajan et al. and Cohen et al., who reported good outcomes in 81.8% and 86.3% of patients, respectively [8,10]. Ten Brinck et al. reported an mRS 0-2 rate of 73.7%, also comparable to our results [10,17].

This study has several limitations. It is retrospective, lacks a control group, includes a small sample size, and was conducted at a single center, which limits the generalizability of the findings. However, it provides a cohort treated with flow diverters, a standardized antiplatelet protocol, and the inclusion of dissecting aneurysms, as well as clinical experience in managing patients treated in a delayed time frame, offering valuable real-world data applicable to similar healthcare systems.

## Conclusions

In this study, the use of flow diverters in ruptured intracranial aneurysms demonstrated a high occlusion rate and favorable functional outcomes, supporting their consideration as a treatment option in carefully selected patients. Despite its retrospective, single-center design and small sample size, this study provides real-world evidence supporting the selective use of flow diversion in appropriately chosen ruptured aneurysms. Larger prospective studies are needed to refine patient selection criteria and determine the optimal timing for treatment.
